# Religion and Mental Health in Racial and Ethnic Minority Populations: A Review of the Literature

**DOI:** 10.1093/geroni/igaa035

**Published:** 2020-08-08

**Authors:** Ann W Nguyen

**Affiliations:** Jack, Joseph and Morton Mandel School of Applied Social Sciences, Case Western Reserve University, Cleveland, Ohio

**Keywords:** Mental health, Racial/ethnic minority elders, Religiosity, Religious participation

## Abstract

Religion has been an important source of resiliency for many racial and ethnic minority populations. Given the salience, sociohistorical context, and importance of religion in the lives of black and Latino Americans, this literature review focuses on the mental health and well-being outcomes of religion among black and Latino Americans across the adult life course and specifically in later life. This review provides an overview of religious participation and religiosity levels and an in-depth discussion of extant research on the relationship between the multiple dimensions of religiosity and mental health in these 2 populations. Racial differences between blacks, Latinos, and non-Latino whites are also examined. Suggestions for limitations of the current literature and future directions for research on religion and mental health in racial/ethnic minority populations, especially older minorities, are proposed.

**Translational Significance:** A better understanding of the mental health effects of religion among older black and Latino adults contributes to more culturally relevant practices and interventions and underscores the intersection of religion and culture in mental health practice.

Research on the link between religion and health has received considerable attention and interest over the last few decades. While the bulk of research in this area examines the association between religion and physical health, fewer studies have systematically investigated the association between religion and mental health. Studies in this area have predominately demonstrated a positive association between religion and mental health and have been prompted by the understanding that religion is a psychological and social resource that can be used to cope with stressors. Moreover, social relationships within religious communities are important sources of social support. Religion has undoubtedly been an important source of resiliency for many racial and ethnic minority populations, and its narrative of perseverance and faith has proven to be a sustaining force for these populations in the face of hardships and inequality. Moreover, religion and religious involvement are deeply embedded in the culture, community life, and group identity of many racial/ethnic minority populations, such as black and Latino Americans. For example, the black church can reinforce group (and ethnic) identity and enhance a person’s sense of self-worth by facilitating social integration within a community that shares similar values, beliefs, and cultural traditions (Mays & Nicholson, 1933). Sermonic traditions based on liberation and defiance theology are also unique to the black church and provide a spiritual framework for coping with discrimination (Frazier & Lincoln, 1974). Thus, it is of no surprise that research has documented the salutary effects of religion on the mental health of black and Latino Americans. The purpose of this literature review is to review extant research on the relationship between religiosity and mental health and well-being among black and Latino Americans. The second aim of this review is to provide a critical analysis of the state of the literature in this area and provide recommendations for further exploration.

Religiosity is a multidimensional concept that encapsulates a range of attitudes, beliefs, and behaviors (Taylor et al., 2004). Research in this area has focused predominately on three dimensions of religiosity—organizational religious participation (i.e., behaviors and practices that are public, institutional, and formal, such as religious service attendance), nonorganization religious participation (i.e., behaviors and practices that are noninstitutional, informal, and conducted in private, such as private prayer), and subjective religiosity (i.e., individual perceptions of being religious). Because most studies of religious involvement and mental health among racial/ethnic minority older adults focus on blacks and Latinos, this will be a selective review of research focusing on these two population groups.

This literature review will predominately focus on older blacks and Latinos and begins within an overview of religiosity among older black and Latino Americans. This section is followed by a discussion of the mechanisms by which religion influences mental health and well-being. The next sections are a review of the connection between religion and mental health and well-being among blacks and Latinos across the adult life course (aged 18 and older, including older adults) and specifically among older blacks and Latinos. This is followed by a review of race comparative studies on religion and mental health and well-being. The negative aspects of religion are presented, and this review concludes with a discussion of the limitations of the extant literature and suggestions for future directions in research on religion in racial/ethnic minority populations. The review of outcomes of religious involvement will focus on diagnosed psychiatric disorders, other mental health concerns (e.g., suicidality and psychological distress), and well-being (e.g., life satisfaction).

## Religiosity Among Older Adults

### Black Americans

For many black Americans, religion is a major part of their lives and an important resource for black families as well as individuals (Taylor et al., 2004). According to the Pew Research Center (2015), 79% of black Americans identify as Christian, and an additional 3% identify with other non-Christian faiths. The black population in the United States is predominately Protestant, with close to half of all blacks identifying as Baptist (49%; Nguyen et al., 2019). In black communities, religion and the church serve many functions above and beyond spiritual sustenance. Historically and contemporaneously, the black church, in addition to being a religious institution, is a social, cultural, civic, educational, and political institution that is central to black communities (Lincoln & Mamiya, 1990). Because of social, economic, and institutional disenfranchisement, black Americans have traditionally had difficulty accessing public and private services. As a result, black churches tend to offer a greater number of community programs and mental health services than white churches (Taylor et al., 2000).

As a group, black Americans have relatively high rates of religious involvement. Three quarters of blacks surveyed by the Pew Research Center indicated that religion is very important in their lives (Pew Research Center, 2015). One in two individuals says that they attend religious services at least once a week (Pew Research Center, 2015). Studies show that religiosity increases with age. Older blacks attend religious services more frequently than younger blacks, and compared to younger blacks, older blacks are less likely to never attend religious services (Chatters et al., 2014; Taylor et al., 2014). In fact, more than half (52%) of older blacks, compared to 38% of younger blacks, attend religious services either nearly every day or at least once a week (Jackson et al., 2004). Older blacks also participate in church activities outside of services more frequently than their younger counterparts (Taylor et al., 2014). Subjective religiosity also varies by age; older blacks are more likely to attribute greater importance to religion in their lives and have a stronger religious identity than younger blacks (Chatters et al., 1999). Finally, the use of clergy to address serious personal problems increases with age (Chatters, Mattis et al., 2011). Taken together, research consistently demonstrates that older blacks have higher levels of religiosity across a number of dimensions than their younger counterparts.

### Latino Americans

Religion and the church have also played a prominent role in Latino communities in the United States. Latino Americans predominately identify as Christian (80%), with Catholics comprising the majority of this population (55%; Pew Research Center, 2013). Protestant, mostly Evangelical, Latinos make up 22% of the Latino population. In Latino American communities, religion facilitates (a) the stress-coping process of migration, (b) reestablishing ethnic communities in the United States, and (c) preservation of cultural values and ethnic identity. Similar to religion in black communities, religion and the church play central roles within Latino communities in the United States (Martinez, 2012; Nabhan-Warren, 2016). Often churches in Latino communities have distinctive ethnic characteristics, such as Spanish language services and Latino cultural programs, and are institutions that help maintain Latino identity. Thus, religious and ethnic/cultural identities are complexly interwoven in Latino communities.

Overall, Latinos demonstrate relatively high levels of religiosity, with 60% indicating that religion is very important in their lives (Pew Research Center, 2013). The overwhelming majority of Latinos (91%) also display moderate to high levels of religious commitment (Pew Research Center, 2013). National survey data indicate that religiosity also increases with age among Latinos. For example, close to three in four Latinos aged 50 and older say that religion is very important in their lives (Pew Research Center, 2013). This is compared to 45% of Latinos aged 18–29 years and 63% of Latinos aged 30–49 years who say that religion is very important. Older Latinos also exhibit higher levels of religious commitment than their younger counterparts (Pew Research Center, 2013). At the organizational level, close to half of older Latinos, compared to nearly one of three young Latinos, attend religious services at least once a week (Pew Research Center, 2013). Older Latinos have higher rates of participation in church activities outside of worship, albeit this difference is only slight (Pew Research Center, 2013). Older Latinos’ rates of nonorganizational religious involvement are also higher than those of younger Latinos. For instance, about three quarters of older Latinos, when compared with less than half of young Latinos, pray at least once a day (Pew Research Center, 2013).

## Mechanisms of Religion

This section will provide a brief overview of some of the scholarship on the pathways between religiosity and mental health and well-being. For more detailed information in this area, please refer to the works of Chatters (2000), Ellison and Levin (1998), and Schieman et al. (2013). Scholarship on the mechanisms by which religion affects mental health and well-being suggests that it can have both direct and indirect effects on these outcomes. Religious activities, such as prayer, reading religious texts, and other forms of worship, can promote positive emotions and a sense of well-being, directly leading to mental health and well-being (Chatters, 2000; Taylor et al., 2004). For example, praying can induce a sense of calm and peace as well as promote positive emotions (Taylor et al., 2004).

Religious involvement can also have an indirect effect on mental health by promoting healthy behaviors (Chatters, 2000; Ellison & Levin, 1998; Levin & Chatters, 1998). Many religions have values and beliefs that promote healthy behaviors, such as abstaining from alcohol and nicotine use. Those who are religiously involved, especially at the organizational level, are embedded within the community that shares these values and beliefs. Thus, religious participation, by virtue of social influence, can promote and reinforce these healthy behaviors. These behaviors can prevent physical health problems, which is particularly important for older adults who are at greater risk of physical ailments due to age. Research on physical morbidities demonstrates a strong positive association between physical and psychological morbidities (Chapman et al., 2005). Consequently, religious participation can indirectly affect mental health by promoting healthy behaviors that prevent physical ailments. For example, research indicates that church support and service attendance are associated with the adoption and maintenance of healthy lifestyles and a decrease in unhealthy behaviors (e.g., smoking; Krause et al., 2011; Strawbridge et al., 2001). A study of Seventh-day Adventists, a denomination that explicitly promotes a healthy lifestyle, especially diet, found that a healthy lifestyle mediates the negative association between religious involvement and all-cause mortality (Morton et al., 2017).

Another way that religion can indirectly influence mental health is by operating as a stress-buffering or stress-coping resource (Chatters, 2000; Ellison & Levin, 1998). For example, many individuals turn to prayer to cope with stressful situations. During times of stress, individuals may pray for guidance or intervention from God in dealing with the stressor (Taylor et al., 2004). Reading religious texts, listening to religious radio programs, and watching religious television programs are examples of other stress-coping strategies. Individuals may engage in these religious media as a means to gain inspiration or guidance in dealing with their problems or as a way to relieve stress and a sense of psychological distress (Taylor et al., 2004).

Congregational relationships are major social resources, as congregants provide the support that is important for dealing with stressors. In fact, some research suggests that congregational support mediates the positive association between service attendance and mental health (Koenig, 1998). In other words, individuals who attend religious services more frequently are more likely to have regular contact and closer ties with congregants and to be more socially embedded within the congregational network (McFarland, 2009; Schieman et al., 2013). These factors facilitate more frequent exchanges of congregational support, which can be used for coping with stressors. Clergy is also an important source of support for churchgoers. Clergy provides a range of support (e.g., advice, encouragement, counseling, and instrumental assistance) and help with a number of problems (e.g., mental health, marital, grief, and material hardship; Taylor et al., 2004). Close, personal relationships with clergy make these helping relationships more meaningful and effective.

It is important to note that, in certain situations, some of the aspects of religious involvement are positively associated with mental health problems. This is likely due to a phenomenon known as resource mobilization. The resource mobilization framework (Wheaton, 1985) posits that as an individual experiences a stressor and its psychological sequelae, they are likely to marshal coping resources to deal with these stressors. For example, a person who is experiencing a personal problem that is causing psychological distress may, in response, turn to prayer or pray more frequently than usual in an attempt to alleviate this sense of distress. Thus, studies that find that positive religious aspects are associated with increased mental health problems may be demonstrating resource mobilization.

Negative aspects of religious involvement, such as negative interaction with congregants, religious doubt, and negative religious coping, are often linked to poorer mental health and well-being. Several pathways are proposed in the current literature linking these negative religious aspects with poor mental health. First, negative religious aspects can exacerbate the effects of stress on mental health (Krause, 2012). For example, spiritual struggles can lead an individual to rely less on religious coping resources and rendering religious coping resources less effective. This amounts to an erosion of coping resources available to the individual that are important for dealing with stressors to attenuate their negative effects.

Second, religion can negatively affect mental health via social relationships and interactions with congregants and clergy (Chatters, 2000). Social relationships within religious communities can be a source of distress when the individual does not conform to religious and institutional expectations and norms. When an individual fails to conform to their religious community’s expectation and norms, members of the community are likely to sanction the individual. Furthermore, negative interaction with church members and clergy, such as interpersonal conflict and criticisms, are also sources of distress (Chatters, 2000). Although they occur less frequently than positive social interactions, negative interactions are perceived as stressors because they are unexpected and violate expectations of civility (Rook, 1990). Moreover, negative interactions are stressful because they contradict expectations and norms for behaviors within a religious setting (Chatters, 2000). As a stressor, negative interaction can both directly and indirectly affect mental health and well-being. Negative interaction can directly lead to distress, negative affect, and other mental health problems. Indirectly, negative interaction can erode a person’s sense of self-worth and mastery (Lincoln, 2007) and hinder effective coping behaviors and psychological functioning (Rook, 1984). These effects can, in turn, result in elevated distress levels and compromised mental health and well-being.

Third, negative religious coping, the negative religiously framed response to a stressor, amounts to maladaptive cognitions framed within the context of religion. Individuals who engage in negative religious coping tend to have heightened perceptions of threat, have a less secure relationship with God, and experience religious doubt and spiritual struggles (Chatters, 2000). These characteristics can contribute to a decreased sense of well-being and mental health.

## Religion and Mental Health Across the Life Course

### Black Americans

Empirical evidence documents the relationship between religion and a broad range of mental health indicators. Religious service attendance, in particular, is strongly associated with mental health among blacks across the life course. Studies on service attendance find that it can protect against a wide range of mental health problems, including depression, substance use disorders, suicide, anxiety disorders, and depressive symptoms (Chatters et al., 2018; Himle et al., 2012; Robinson et al., 2012; Taylor et al., 2011). Congregational relationships are also important for mental health. For example, individuals who are in more frequent contact with church members report fewer depressive symptoms (Chatters et al., 2018). Research on church relationships and suicide finds that frequency of contact with and emotional closeness to church members are both related to decreased suicidality (Chatters, Taylor et al., 2011).

Research also identifies subjective religiosity and nonorganizational religious involvement as protective factors for mental health. blacks who report higher levels of subjective religiosity have less psychological distress (Jang & Johnson, 2004; Levin & Taylor, 1998) and a decreased likelihood of having suicidal ideation (Taylor et al., 2011). Furthermore, the use of religious guidance is associated with lower odds of major depression (Ellison & Flannelly, 2009). Religious coping strategies, such as reading religious texts and looking to God for strength, are also associated with better mental health (Taylor et al., 2011, 2012). However, some studies find that reading religious texts is associated with an increased odds of suicidal ideation and depression (Taylor et al., 2011, 2012), and other forms of nonorganizational religious participation (e.g., prayer and meditation) are predictive of more depressive symptoms (Ellison, 1995) and higher odds for meeting criteria for obsessive-compulsive disorder (OCD) (Himle et al., 2012).

### Latino Americans

Of the few studies that have investigated the relationship between religion and the mental health of Latino adults, the majority of these studies have focused on service attendance and subjective religiosity. Research on service attendance finds that it is associated with decreased odds for depressive, anxiety, and substance use disorders (Moreno & Cardemil, 2018). Additionally, more frequent service attendance is inversely associated with suicidal ideation and attempts (Robinson et al., 2012) and depressive symptoms (Ellison et al., 2009). A study that examined the mental health of Latino caregivers reported that caregivers who attended religious services more frequently and had higher levels of organizational religiosity were less likely to have depression (Sun & Hodge, 2014).

Among Latino Americans, higher subjective religiosity is associated with lower odds of having a lifetime, 12-month, and past-month substance use disorder (Becerra et al., 2014) and suicidal ideation (Hovey, 1999), fewer depressive symptoms (Ellison et al., 2009), and less anxiety (Hovey & Magaña, 2002a, 2002b, 2002c). Evidence for the use of religion as a stress-coping resource shows that Latinos who engage in positive spiritual and religious coping are less likely to be depressed (Sun & Hodge, 2014). Prayer, another important coping strategy, is negatively associated with depression among Latino caregivers (Sun & Hodge, 2014). Research on seeking spiritual comfort indicates that Latinos who report that they sometimes seek spiritual comfort have more depressive symptoms than Latinos who report that they either rarely or never seek spiritual comfort (Ellison et al., 2009). This is consistent with studies that have found that some aspects of religious involvement have a nonlinear relationship with mental health and well-being (Krause, 1995; McFarland, 2009). That is, individuals with high levels of religious involvement or no religious involvement have better mental health than individuals with low or moderate levels of religious involvement. Low to moderate levels of religious involvement may reflect uncertainty in religious beliefs and faith, which is associated with poorer mental health (Krause, 2012).

## Religion, Mental Health, and Well-Being Among Older Adults

### Older Black Americans

#### Mental health outcomes

Empirical evidence indicates that multiple religiosity dimensions are consistently negatively associated with a range of mental health problems among older blacks. Findings demonstrate that older blacks who report more frequent service attendance are less likely to have a mood disorder and other psychiatric disorders (Chatters et al., 2008). Additionally, more frequent service attendees meet the criteria for a fewer number of psychiatric disorders than less frequent service attendees. Krause (2003b) reported that more frequent service attendance was associated with both less depressed affect and somatic depressive symptoms. A study that examined service attendance specifically among older blacks with cancer found that patients who reported more frequent service attendance also reported more positive affect (Musick et al., 1998). Research on subjective religiosity indicates that blacks who report higher levels of subjective religiosity are less likely to meet criteria for a lifetime DSM-IV psychiatric disorder and are likely to meet criteria for a fewer number of psychiatric disorders than blacks who report lower levels of subjective religiosity (Chatters et al., 2008).

Studies on church relationships indicate that receiving emotional support is associated with fewer depressive symptoms and lower levels of serious psychological distress (Chatters et al., 2015). In addition to its protective qualities, research has also identified church support as a moderator in the association between discrimination and distress. An investigation found that, among men in late adulthood, the positive association between discrimination and serious psychological distress was more pronounced among men who reported receiving more frequent support from church members than among men who reported receiving infrequent support (Nguyen et al., 2017). Another study that examined the moderating effects of church support in a nationally representative sample of older blacks found that more frequent contact with and higher levels of emotional closeness to church members moderated the negative effects of discrimination on generalized anxiety disorder (GAD; Nguyen, 2018). Specifically, this study found that although more frequent experiences of discrimination were associated with greater odds of meeting criteria for GAD among those who reported low levels of contact and support from church members, discrimination was unrelated to GAD among those who reported high levels of contact and support from church members.

#### Well-being outcomes

Empirical evidence on well-being among older blacks indicates that religion is associated with a range of well-being outcomes. In a sample of older blacks living in New York City, researchers found that those who had higher rates of organizational religious participation reported higher levels of environmental mastery, personal growth, self-acceptance, and purpose in life (Frazier et al., 2005). These researchers also found that nonorganizational religious participation was related to higher levels of personal growth, self-acceptance, and purpose in life. Studies on subjective religiosity indicate that it is predictive of higher self-esteem (Krause, 1992), environmental mastery, personal growth, purpose in life, and self-acceptance (Frazier et al., 2005). Religious meaning is associated with higher levels of life satisfaction, self-esteem, and optimism (Krause, 2003c). Furthermore, multiple facets of church relationships are linked to well-being in this population. For example, higher levels of perceived overall support from church members are associated with greater well-being (Walls & Zarit, 1991). Receiving more frequent emotional support from congregants is associated with greater life satisfaction (Krause, 2004), and receiving more frequent emotional support from clergy is associated with greater feelings of self-worth (Krause, 2003a). Finally, a study examining religion and well-being in rural older adults found that older blacks who engaged in religious and spiritual coping strategies were more likely to report higher levels of life satisfaction (Yoon & Lee, 2004).

### Older Latino Americans

#### Mental health outcomes

Research on religion and mental health that focus exclusively on older Latinos is severely limited. The handful of studies that have focused on this population has predominately focused on service attendance. Using data from the Hispanic Established Populations for Epidemiological Studies of the Elderly, a population-based study of community-dwelling Mexican Americans aged 65 and older residing in five Southwestern states, researchers found that frequent service attendees had fewer depressive symptoms than infrequent service attendees (Reyes-Ortiz et al., 2008). A second study using the same data set focused on service attendance and changes in depressive symptoms during widowhood (Monserud & Markides, 2017). This study found that although older Mexican American widows/widowers reported higher levels of depressive symptoms during the transition to widowhood than pre-widowhood, those who attended church more frequently experienced less steep increases in depressive symptoms in widowhood. Additionally, Aranda (2008) found, in a sample of Latinos aged 50 and older living in the greater Los Angeles area, that respondents who attended religious services more frequently were less likely to report depression.

#### Well-being outcomes

Empirical evidence indicates that religion can also promote well-being among older Latinos. Research on organizational religious participation shows that greater involvement in formal church activities is associated with greater life satisfaction (Krause & Bastida, 2011a). Regarding service attendance, Levin et al. (1996) found in a sample of older Mexican Americans in the San Antonio, Texas area that respondents who attended religious services more frequently reported higher levels of life satisfaction. Similarly, in a national sample of Mexican American, Cuban American, and Puerto Rican American older adults, Angel and Angel (1992) reported a positive association between service attendance and life satisfaction. Other studies on religion among older Latinos identify extrinsic religiosity (i.e., a religious orientation that is characterized by the use of religion and religious behaviors as a means to other ends rather than an end in itself) and spirituality as a correlate of well-being (Hilton & Child, 2014). One particular study found that extrinsic religiosity is associated with less well-being, while spirituality is associated with greater well-being (Hilton & Child, 2014).

Taken together, the cumulative body of empirical evidence indicates that most aspects of religiosity are associated with better mental health and well-being for both minority adults across the life course as well as minority older adults. These studies support the theoretical literature on the mechanisms of religion, which hypothesizes that varied aspects of religiosity have direct and indirect effects on mental health and well-being. Theoretical works conceptualize religion as psychological and social resources that can be used for stress coping and buffering.

## Racial Differences in Religion and Mental Health and Well-Being

### Mental Health Outcomes

Mounting evidence suggests that the salutary effects of religion are stronger for blacks than for whites. For example, an investigation of religion and depression among older adults found that while black respondents who attended church more frequently saw a greater decline in somatic depressive symptoms, church attendance was unrelated to somatic depressive symptoms among white respondents (Krause, 2003b). This investigation also identified race differences in the relationship between service attendance and depressed affect. Service attendance was not associated with depressed affect among white respondents. In contrast, for black respondents, more frequent service attendance predicted greater declines in depressed affect. A study examining the stress-buffering effects of religion using data from the Midlife in the United States (MIDUS) study reported that while more frequent experiences of discrimination were related to higher levels of psychological distress for both black and white respondents, more frequent service attendance attenuated this association but only for black respondents (Bierman, 2006). Research examining racial differences in emotional support from church members among older black and white adults found that while church support was associated with decreased depressive symptoms for both black and white respondents, this association was stronger among black respondents (Assari & Lankarani, 2018)

### Well-Being Outcomes

Turning to well-being, Krause (2003c) found that higher levels of religious meaning were associated with greater life satisfaction, self-esteem, and optimism for both older blacks and whites. However, these associations were stronger for blacks than for whites, indicating that older blacks benefit more from religious meaning than their white counterparts. Another study that examined optimism found even greater racial differences. Krause’s (2002) examination of religion and race among older adults indicated that more frequent service attendance predicted higher levels of optimism for black respondents, but service attendance was inversely related to optimism among white respondents. That is, older whites who attended religious services more frequently reported lower levels of optimism. Finally, extant research indicates that receiving more emotional support from clergy is associated with greater feelings of self-worth among older blacks but clergy support is unassociated with feelings of self-worth among older whites (Krause, 2003a).

These racial differences may be attributable to racial differences in the organization and programmatic emphasis on religious services and, more generally, churches (Assari & Lankarani, 2018). black and white churches were founded upon different missions (Assari & Lankarani, 2018). Historically, the church has represented a safe space, separate from the oppressive environment of a greater society, for expression, healing, and validation for black Americans (Schieman et al., 2013). In particular, sermonic traditions that are based on liberation and defiance theology are unique to black churches and provide an especially relevant spiritual framework for stress coping (Frazier & Lincoln, 1974). Furthermore, as a religio-cultural institution that is primarily founded, financed, and controlled by black communities, the black church can reinforce racial/ethnic identity and promote a greater sense of self-worth, which is an important psychological resource (Mays & Nicholson, 1933). Furthermore, it provides black Americans opportunities for leadership, which can enhance an individual’s sense of self-worth, that would have been difficult to access within the broader society due to disenfranchisement (Myrdal, 1944). Additionally, racial differences in the association between church support and mental health and well-being may be due to differential access to and mobilization of social resources (Assari & Lankarani, 2018). That is, blacks may be more adept at effectively mobilizing their congregational and clergy support networks for stress coping than whites, as reliance on informal sources of support is common in black communities. Studies on racial differences in church support also indicate that black congregants engage in more frequent exchanges of support with each other and receive support from clergy more frequently than white congregants (Krause, 2016; Krause & Bastida, 2011b; Krause & Hayward, 2014; Taylor et al., 2013). These findings suggest that compared to whites, blacks have greater access to social resources within their religious communities that can be used for coping with stress.

## Negative Side of Religion

Although religion is associated with a wide range of mental health benefits, some aspects of religious involvement are harmful to mental health and well-being. Negative interaction with church members is associated with a variety of mental health problems (Chatters et al., 2015, 2018). For example, Chatters et al. (2018) found that black respondents who reported more frequent negative interactions with congregants had more depressive symptoms. Another investigation indicated that more frequent negative interaction with church members was associated with more depressive symptoms and psychological distress among older blacks (Chatters et al., 2015). Among blacks who have experienced discrimination, negative interaction with congregants is associated with a greater likelihood of self-blame for these discriminatory experiences (Hayward & Krause, 2015). Evidence for the impact of negative interaction among older Latinos indicates a similar pattern. Older Mexican Americans who report more negative interaction with congregants also reported more somatic depressive symptoms (Krause & Hayward, 2012).

Religious doubt is another aspect of religion that negatively affects mental health (Krause, 2012). Older Latinos who report more religious doubt have higher levels of depressed affect (Krause & Hayward, 2012) and cognition (Krause, 2012) and greater somatic depressive symptoms (Krause & Hayward, 2012). Among older blacks, religious doubt is also associated with more depressive symptoms (Willis et al., 2019). Similarly, spiritual struggles are predictive of anxiety and less happiness among older blacks and Latinos (Krause et al., 2018). Research on race differences indicates that while spiritual struggles negatively affect the mental health and well-being of all older adults, the strength of its effects is not equal across all racial/ethnic groups (Krause et al., 2018). Krause et al. (2018) found that the associations between spiritual struggles and anxiety and decreased happiness were weaker for older blacks than older whites and Latinos. Lastly, negative religious coping (maladaptive coping strategies such as redefining stressors as punishment from God) can contribute to poor mental health. Among Latinos, negative religious coping is associated with depression and greater psychological distress and exacerbates the positive association between acculturative stress and psychological distress (Herrera et al., 2009; Silva et al., 2017).

## Limitations and Future Directions

In my review of the scholarship on religion and mental health among racial/ethnic minorities, especially older minorities, I have identified several critical gaps in knowledge that future research should address. First, there is a dearth of research on diverse racial/ethnic minority groups. While the major of minority research in this area is on black Americans, there are far fewer studies on Latino Americans and only a handful of studies on other minority groups, such as Native Americans and Asian Americans, especially in gerontology. Given this, little is known about the effects of religion on the mental health of these populations. The rapidly expanding older adult population in the United States is also increasing in racial/ethnic diversity (Ortman et al., 2014). Thus, a better understanding of the effects of religion in diverse populations is of critical importance. In particular, although U.S.-born Latinos comprise a sizeable proportion of the Latino American population, much of the research on Latino Americans is focused on immigrants, as evidenced by the studies reviewed in this article. U.S.-born and immigrant Latino Americans differ on a number of characteristics (e.g., religious affiliation/denomination, educational attainment, and language) that may influence the nature of their religious involvement and mental health. Furthermore, the Latino American population is racially diverse and comprises people with black, white, Asian, indigenous, and mixed ancestry. However, the majority of studies that focus on Latinos do not make racial distinctions, and research often treats Latinos as a single, racially homogeneous group. Thus, it is important that future research takes into consideration the within-group variability of older Latino adults. In addition to nativity, research on older Latinos should also examine within-group differences related to race, nationality, and immigration pathways (e.g., voluntary immigrant vs. refugee). More nuanced research on diverse minority populations and the inclusion of diverse minority groups in survey research are necessary to address this limitation.

Second, studies on religion and mental health, especially those on older Latinos, predominately focus on the effects of service attendance. Religiosity comprises multiple dimensions and a broad range of behaviors, attitudes, and beliefs, which are not fully captured with a limited focus on service attendance. Future research should examine a broader range of religious involvement measures, such as church relationships (e.g., supportive exchanges and negative social interaction with church and clergy members), religious meaning, and use of religious coping. Extending research beyond a focus on service attendance is important because research indicates that service attendance is likely indirectly associated with mental health and is mediated by a number of other religious involvement factors. For instance, church support may mediate the association between service attendance and mental health (Schieman et al., 2013); individuals who attend religious services more frequently are likely to have more frequent contact with church members and be more socially integrated within the congregational network. These factors, in turn, can lead to more frequent support exchanges with church members, which are associated with better mental health and well-being. Consequently, future research should examine a broader range of religiosity measures to gain a more nuanced understanding of how various aspects of religious involvement are related to mental health and how they may mediate the relationship between service attendance and mental health.

Third, Latino Americans have a diverse range of religious practices and traditions, such as making mandas (pleas to the Virgin Mary or saints) and juramentos (ritualized pledges), that could potentially influence their mental health. Yet, with the exception of a few studies, the majority of the investigations of religion and mental health in this population have focused on service attendance and subjective religiosity. Future studies should consider common religious practices and traditions that are specific to Latinos, which can yield more culturally relevant research and practice implications and underscore the intersection of religion and culture in mental health.

Fourth, although the theoretical literature has proposed numerous pathways/mechanisms by which religion influences mental health and well-being, very few studies have investigated these mechanisms. The majority of studies on the religion–mental health connection tests associations between various aspects of religious involvement and mental health outcomes based on implicit assumptions of the underlying mechanisms of religion rather than directly and systematically test these mechanisms. Thus, much of our knowledge on the mechanisms of religion remains theoretical and is yet to be confirmed. Studies that explicitly investigate the mechanisms that are proposed in the theoretical literature, such as biological, psychological, and social mechanisms, are necessary to develop a more complete empirical picture of the functions of religion. Additionally, future research should also examine how these causal pathways may differ between racial/ethnic groups and over the life course to determine between- and within-group differences.

Fifth, there is a dearth of longitudinal studies that investigate the association between religion and mental health. Longitudinal studies would provide clarification for the mechanisms by which religion influences mental health. Causal interpretations are not possible with a cross-sectional design. For example, it is unclear whether studies that have found positive associations between religious involvement and poor mental health (Ellison, 1995; Himle et al., 2012; Taylor et al., 2011, 2012) are demonstrating that religious involvement in some contexts can be harmful to mental health. Alternatively, these studies may indicate resource mobilization, in which distressed individuals marshal their coping resources (e.g., religiosity) to deal with stressors. Longitudinal studies are particularly helpful for interrogating these kinds of findings as well as the causal mechanisms of religion.

Finally, there is a lack of nationally representative data sets with (a) a sizeable older minority sample, (b) diverse measures of religiosity, and (c) extensive and robust measures of mental health/illness. Many of the studies discussed in this review relied on regional samples, which have limited generalizability. Although the Health and Retirement Study, which is one of the most well known and widely used national panel study data sets of older adults, oversamples for black and Latino respondents, its measures of religiosity and mental health are relatively limited. Other national older adult data sets that include some measures of religiosity and mental health, such as the MIDUS study, National Health and Aging Trends Study, and National Social Life, Health, and Aging Project, have relatively small minority samples, which presents issues of statistical power, especially for more complex analyses and within-group study designs. To my knowledge, the National Survey of American Life: Coping with Stress in the 21st Century (NSAL) is the only national data set that has a large black American (African American and black Caribbean) sample, extensive measures of religiosity (e.g., use of clergy, church relationships, negative interaction with congregants, work at church, participation in church activities, and religious media consumption), and a diverse range of mental health variables (e.g., major depressive disorder, posttraumatic stress disorder, GAD, OCD, and substance use disorder) assessed via structured diagnostic interviews that align with the diagnostic criteria of the DSM-IV. However, because the NSAL is a data set of adults aged 18 and older, the older black sample is relatively smaller (*N* = 1,141). The lack of nationally representative data is a contributing factor to all of the limitations previously discussed in this section. More nationally representative data are necessary to bridge these knowledge gaps. This can be addressed by the development of national studies that include (a) large and racially/ethnically diverse older adult samples (e.g., Asian Americans, Native Americans and Alaskan Natives, and Native Hawaiians and Pacific Islanders) and (b) explore a broad range of religiosity and mental health variables. Alternatively, amendments to ongoing national studies could be made to address the inclusion of diverse racial/ethnic groups and incorporate a broad array of religious involvement and mental health and well-being indicators.

### Practice Implications

Clergy is another important source of support for blacks. The use of clergy for mental health problems is relatively common, and for some individuals, clergy is their primary resource for mental health problems (Anthony et al., 2015; Hankerson et al., 2015; Stansbury et al., 2018). Clergy and the church are well situated to address the mental health needs of this population for several reasons. A qualitative study of pastoral care in black churches indicated that clergy members tend to have a holistic view of mental health—mental health, spirituality, and physical health are inseparable (Stansbury et al., 2011). In fact, church-based interventions are often culturally tailored to emphasize black culture and spirituality (Hankerson & Weissman, 2012). Counseling informed by this holistic perspective may be more consistent with the values and beliefs of the black community. Furthermore, the church is viewed as a safe space for discussing mental health concerns (Campbell & Winchester, 2020). These qualities can address the barriers to service utilization (e.g., stigma and mistrust of mental health professionals) that have contributed to the underutilization of formal mental health services by black Americans (Hankerson et al., 2018; Hankerson & Weissman, 2012). However, not all members of the clergy are knowledgeable in and/or trained to work with congregants on mental health issues (Anthony et al., 2015). In instances in which clergy members lack mental health expertise, referrals to community mental health centers are often made (Stansbury, 2011).

black and Latino churches are uniquely positioned to respond to the specific mental health needs of older black and Latino adults. Collaborations between black and Latino churches and community mental health agencies can expand access to mental health care for traditionally underserved communities. In particular, collaborations with clergy, who act as gatekeepers to church members, are especially important (Adksion-Bradley et al., 2005). Close relationships between clergy and mental health service agencies can increase referrals to formal, professional mental health care, especially in instances in which clergy members lack adequate mental health training. An example of such a collaborative strategy is the designation of a mental health professional to serve as a liaison to the church and clergy (Adksion-Bradley et al., 2005). As a liaison, the mental health professional could familiarize church members with the services offered by community agencies and the process of obtaining these services. The advantage of partnering with clergy is that it signals to congregants that secular mental health care is consistent with the church’s values and beliefs, the importance of addressing mental health/illness, and, to some extent, normalizes mental illness and professional mental health care.

Given the importance and relevance of church members, congregational relationships should be considered in clinical practice with older black and Latino clients. For example, initial clinical assessments should assess clients’ level of religious involvement and relationships with church members. In particular, information on objective (e.g., network size and frequency of contact) and subjective (e.g., satisfaction with relationships and subjective closeness) relationship qualities should be obtained to help determine the client’s available stress-coping resources. Additionally, interventions to increase formal mental health service use among older minorities should consider the role of church members. Interventions to increase formal mental health service use among older minorities could train select church members, especially those who are socially well connected within the church and are able to reach a greater number of congregants, to (a) provide psychoeducation on available community mental health services and their benefits; (b) identify available mental health services that are appropriate for individuals experiencing specific common mental health problems (e.g., depression and anxiety), and (c) provide guidance on accessing mental health services. Overall, the importance and relevance of religion and the church in the lives of older black and Latino Americans suggest that mental health professionals and agencies should consider collaborations with churches and religious communities in order to provide more accessible and culturally relevant services.
